# Sentence Context Prevails Over Word Association in Aphasia Patients with Spared Comprehension: Evidence from N400 Event-Related Potential

**DOI:** 10.3389/fnhum.2016.00684

**Published:** 2017-01-10

**Authors:** Elvira Khachatryan, Miet De Letter, Gertie Vanhoof, Ann Goeleven, Marc M. Van Hulle

**Affiliations:** ^1^Laboratory of Neuro- and Psychophysiology, Department of Neuroscience, KU LeuvenLeuven, Belgium; ^2^Neurology Department, Yerevan State Medical UniversityYerevan, Armenia; ^3^Department of Speech, Language and Hearing Sciences, Ghent UniversityGhent, Belgium; ^4^Department of Speech and Language Therapy, University Hospitals LeuvenLeuven, Belgium; ^5^Department of Neuroscience, KU LeuvenLeuven, Belgium

**Keywords:** aphasia, event-related potentials, lexical association, N400, comprehension deficit, P600

## Abstract

Behavioral and event-related potential (ERP) studies on aphasia patients showed that lexical information is not lost but rather its integration into the working context is hampered. Studies have been conducted on the processing of sentence-level information (meaningful versus meaningless) and of word-level information (related versus unrelated) in aphasia patients, but we are not aware of any study that assesses the relationship between the two. In healthy subjects the processing of a single word in a sentence context has been studied using the N400 ERP. It was shown that, even when there is only a weak expectation of a final word in a sentence, this expectation will dominate word relatedness. In order to study the effect of semantic relatedness between words in sentence processing in aphasia patients, we conducted a crossed-design ERP study, crossing the factors of word relatedness and sentence congruity. We tested aphasia patients with mild to minimum comprehension deficit and healthy young and older (age-matched with our patients) controls on a semantic anomaly judgment task when simultaneously recording EEG. Our results show that our aphasia patient’s N400 amplitudes in response to the sentences of our crossed-design study were similar to those of our age-matched healthy subjects. However, we detected an increase in the N400 ERP latency in those patients, indicating a delay in the integration of the new word into the working context. Additionally, we observed a positive correlation between comprehension level of those patients and N400 effect in response to meaningful sentences without word relatedness contrasted to meaningless sentences without word relatedness.

## Introduction

Aphasia is one of the most common neurological syndromes as up to 35% of post-stroke patients suffer from it Wade et al. (1986), Kauhanen et al. (2000). Aphasia is an impairment in the ability to formulate, express and/or comprehend written and/or spoken language (Freitas, 2012). During the first 3–6 months post-onset, a spontaneous recovery of linguistic abilities can be observed (Lendrem and Lincoln, 1985), but thereafter the odds of spontaneous improvement are very low (Basso et al., 1982). Although, aphasia includes the problem of comprehension and/or production of both spoken and written language, the majority of aphasia studies on comprehension focus on the auditory modality only (Aerts, 2014).

Patients with aphasia use a number of strategies for authentic reading (up to 28 in 3 patients with mild impairment discussed in Lynch et al., 2013) with the aim to improve the following functions: efficiency, contextualization, comprehension, and socialization. Some of those strategies might still lead to erroneous comprehension as in the case of grammatical reduction, during which the close class words (prepositions, determiners, conjunctions, and so on) are usually omitted, or the use of visual analogy and automaticity when reading aloud, during which the patient is looking for links between presented words. This is a big issue as reading deficits of an otherwise relatively recovered individual could seriously hinder a successful return to professional and social life.

One of the methods currently used for investigating language comprehension in aphasia patients is the event-related potential (ERP) – an EEG component time-locked to the presentation of a stimulus of interest (Luck, 2005). When investigating semantic processing in those patients, particularly sentence semantics, many ERP studies rely on the auditory presentation of their stimuli (Swaab et al., 1997, 1998). In our ERP study on aphasics, we investigate semantic processing in sentence comprehension using written stimuli. The main ERP that is believed to reflect the processing of a potentially meaningful stimulus (congruent sentence or discourse, associatively- or semantically related word pairs) is the N400, a negative going potential that in young, healthy subjects starts around 250 ms after presentation of the stimulus of interest (in this case, a single word) and lasts till around 500 ms with a peak around 400 ms (whence its name). Its amplitude increases with the degree of incongruency of sentences or discourses or unrelatedness of word pairs (Kutas and Federmeier, 2011). There are several theories about the significance of the N400 ERP: an index of the difficulty of retrieving word meaning from semantic memory (Lau et al., 2008), the integration of word meaning into the working (active) context and general world knowledge (Hagoort et al., 2004), the violation of the expectation of an upcoming word based on the active context (Kutas and Hillyard, 1980; Dambacher et al., 2006), etc. Another point of discussion is whether the N400 represents lexical- (Deacon et al., 2000; Lau et al., 2009) or post-lexical processing of a word (Brown and Hagoort, 1993; Daltrozzo et al., 2012).

The N400 potential has been repeatedly studied in patients with aphasia (Hagoort et al., 1996; Swaab et al., 1997; D’Arcy et al., 2003; Kojima and Kaga, 2003; Wassenaar and Hagoort, 2005). The amplitude of the N400 was shown to highly correlate with neuro-linguistic test scores, whence showing its potential for assessing language abilities in patients with aphasia independently of behavioral measurements (D’Arcy et al., 2003).

Some ERP studies on semantic incongruity in sentence comprehension showed that aphasics with mild or no comprehension deficit, established with the traditional aphasia test [e.g., Aachen Aphasia Test (AAT)], had an N400 potential in response to incongruent sentences similar to healthy age-matched controls (in terms of both amplitude and latency). Those with moderate or severe comprehension deficit showed either diminished or delayed N400 potentials in response to incongruent sentences (Swaab et al., 1997). Hagoort et al. (1996) showed that, when presented with a semantic- or associative-priming paradigm, the N400 potential (both amplitude and latency) of patients with very mild comprehension deficit was similar to that of healthy subjects while the N400 amplitude of patients with severe comprehension deficit was significantly reduced. Here, as well as in Swaab et al. (1997), using semantically meaningful and meaningless sentences, they concluded that the severe comprehension deficit was a consequence of an impaired integration of individual word meaning into the working context, rather than the loss of lexico-semantic information. Additionally, a number of behavioral (Grindrod and Baum, 2003) and ERP studies (Swaab et al., 1998), showed that, although lexical access is generally spared in aphasia patients, the lexical/contextual selection, hence, the integration of ambiguous words into sentence context is delayed. Unlike the mentioned studies, Kawohl et al. (2010), using visually presented short sentences, showed that aphasic patients with high level of comprehension had a delayed N400 in response to incongruity. Furthermore, patients with severe comprehension deficit showed no N400 potential in response to incongruent sentences. They again suggested that this potential represents a reflection of semantic integration, which is delayed in aphasic patients (including the ones with mild comprehension deficit), and that word processing in patients with severe comprehension deficit differs from patients with mild comprehension deficit. Additionally, they showed that in those patients, repetition of a word does not play a significant role in processing incongruent sentences.

All mentioned studies suggest a delay or difficulty of integration into the active context or a disturbance in lexical access in aphasia patients rather than a loss of lexical-semantic information. But what about word integration in sentence context in case of conflicting word- and sentence – level information?

Although, in Swaab et al. (1997) comprehension of semantically incongruent sentences was studied and in Hagoort et al. (1996) shown that aphasia patients are still able to process both semantically and associatively related word-pairs, albeit with increased difficulty for those with more severe comprehension deficit, we are not aware of any study that investigated how word-associations are processed in sentence context in aphasia patients. In light of the strategies mentioned above, we believe it is important to investigate this topic in order to understand whether it leads to an increased value of word association in sentence processing in patients with aphasia. In healthy individuals, sentence processing depends on the lexical characteristics of the read word in the sentence (word frequency, word length and orthographic neighborhood size), lexico-semantic relationships between the words (lexico-semantic associations) and the expectation of the upcoming word (Van Petten and Kutas, 1990; Van Petten, 1993; Hoeks et al., 2004). It was shown that, when the expectation of the upcoming word is high enough, it will completely over-ride the influence of lexical information. A clear example of expectation generation is for the last word of a sentence: for healthy subjects, it has been shown that sentence context can easily override word association information (Van Petten et al., 1999; Khachatryan et al., 2014) when contrasting final words of congruent sentences with incongruent ones. Moreover, even if there is a weak expectation of the final word in a sentence, those expectations rather than word-association decide the level of integration of the final word into the sentence context (Khachatryan et al., 2014). Conversely, when word association was able to partially or completely modulate sentence processing (Van Petten, 1993; Kuperberg et al., 2003; Hoeks et al., 2004), instead of an increased N400 amplitude in response to incongruent sentence with associations, a later positive ERP component, called Late Positive Complex (LPC) or P600 (as it occurs around 600 ms), was modulated. Here, the P600 in response to incongruent sentences with associations was more positive compared to other sentences. It was suggested that this component reflects the organization and update of the mental representation of incoming information (Brouwer and Hoeks, 2013) and, when word level information was in conflict with sentence level information, the effort to “fix” the mistake and integrate the word into the active context (Kuperberg et al., 2003; Hoeks et al., 2004). Daltrozzo et al. (2012) by using audio presentation of masked sentences showed that P600 reflects post-lexical controlled processes, rather than automatic lexical ones.

Coulson et al. (2005) advanced the investigation of word-association processing in sentence context by conducting a study on healthy young graduate students using different visual hemifield presentations and a crossed lexico-semantic priming paradigm (crossing factors of word-association and sentence congruity). They observed some degree of hemispheric asymmetry for word-association processing in sentence context. They embedded related and unrelated word-pairs into congruent and incongruent sentences (the word-pairs were the last words of the sentence), and presented them in the left or right visual hemifield. According to them, in this case, for some short period (enough to elicit the N400), information is processed only in one hemisphere. They showed that when related words embedded in incongruent sentences are presented to the right visual hemifield (left hemisphere), a small effect of association is observed. However, when presentation was switched to the left hemifield (right hemisphere), this effect was observed in congruent sentences. Hence, one expects that in aphasia patients, whose left hemisphere is impaired, the effect of word-association should also be observable.

Based on the Lynch et al. (2013) study on reading strategies and the one of Coulson et al. (2005) with lateralized presentation of lexico-semantic stimuli, we hypothesize that in aphasic patients word association might play a more significant role in comprehension of sentence level information than in healthy subjects. This might even be evident in patients with mild comprehension deficit (our target patient group). Additionally, based on previous N400 studies on aphasics (Kawohl et al., 2010), we expect the amplitude and/or latency of the N400 potential of these patients to be different from healthy controls.

In order to address our hypothesis, we conducted an ERP experiment on aphasia patients with mild comprehension deficit using a crossed-design paradigm, where relatedness^1^ between words (semantic and/or associative) and sentence congruity factors are crossed. We tested two control groups (young and older adults, the latter age-matched to our patients) to account for the effect of age on the N400 ERP, as it was shown that N400 amplitude and/or latency changes with age (Federmeier and Kutas, 2005; Faustmann et al., 2007). If our hypothesis is correct, and word- association plays a prominent role in processing sentence-level information in those patients, then the N400 potential amplitude should be larger in response to sentences without associations between prime and target words irrespective of or in addition to sentence congruency. Otherwise, the N400 potential should not be different for sentences with or without associations as it was shown to be the case with healthy subjects (Van Petten et al., 1999).

## Materials and Methods

### Participants

A group of 20 healthy young graduate and undergraduate students [average age 20.95 years, standard deviation (SD) 1.9 years, nine females, four left handed], a group of 15 aphasia patients [average age 56.6 (*SD* = 12.0) years, six females, two pre-morbid left-handed] and 12 healthy older subjects [average age 52.5 (*SD* = 5.7) years, 10 females, one left handed] participated in the study. All participants had Dutch as their mother tongue. The average post-onset time for the patients was 27.1 [standard error (SEM) = 8.5] months. The cause of aphasia in 12 out of 15 patients was ischemic stroke, one patient had hemorrhagic stroke and two suffered from a hemorrhagic transformation of an initially ischemic stroke. Results from the Dutch version of the AAT (Graetz et al., 1991) for 13 of the patients and the lesion location of all 15 patients are listed in **Table 1**. Two patients (M. I. and S. L.) did not have results on AAT; hence, we did not include them in some of the reported analyses. The diagnosis of aphasia was established by a speech and language therapist according to the Boston Classification of Aphasia (Goodglass and Kaplan, 1983). Five patients suffered from amnestic aphasia, four from Broca, four from Wernicke, one from transcortical motor- and one from transcortical sensory aphasia. Unlike patients, both young and older control subjects were paid for their participation. Patients were recruited from the Leuven University Hospitals and Ghent University Hospitals. All subjects had normal or corrected to normal vision, none of them reported a history of epileptic seizure or any neurological or psychiatric condition different from the one of interest (i.e., stroke that caused aphasia). None of them were on anti-epileptic or psychotropic medication. The study was conducted according to the latest version of Declaration of Helsinki, following ethical approval from our University Hospitals’ Ethical Committee. Before the experiment and after being informed about its set-up and goal, all participants gave their written consent for the participation.

**Table 1:** Lesion locations and results of subcomponents of AAT for patients (in percentiles).

Patient	Lesion location	AAT subcomponents				
		
		TT	Rep.	Wr.	Nam.	Comp.
H. P.	Left temporoparietal	98	91	99	99	99
L. V.	Left frontoparietal	45	76	70	96	84
D. S.	Right frontotemporal and lentiforme nucleus	100	100	100	100	99
P. I.	Left caudate nucleus and lentiforme nucleus + temporal and inferior frontal gyrus + insula	74	52	99	82	90
V. L.	Left insula + frontotemporal opercula + putamen + caudate nucleus	94	84	100	98	99
B. A.	Left caudate nucleus + capsula interna + lentiforme nucleus	100	97	90	97	75
L. R.	Left frontal gyrus + caudate nucleus + insula	69	99	100	97	99
B. H.	Left parietotemporal	57	76	94	92	99
S. J.	Left parietotemporal	70	98	99	100	99
C. A.	Left temporal	63	85	100	98	99
J. D.	Left parietotemporal	97	91	93	99	99
E. N.	Left fronto-temporoparietal	67	84	99	68	83
E. J.	Left fronto-parietal	85	58	76	50	97
S. L.	Left fronto-temporoparietal	–	–	–	–	–
M. I.	Left fronto-parietal	–	–	–	–	–


*TT, token test; Rep., repetition; Wr., writing; Nam., naming; Comp., comprehension.*

### Materials

Two hundred eighty semantically correct (congruent) and incorrect (incongruent) sentences were used (equally divided). Sentences were partly composed by the authors and partly adapted from Swaab et al. (1997) and Hagoort (2003). Before using these sentences in our EEG experiment, a written survey was administered to 40 graduate and undergraduate students asking them to complete the stem (i.e., the whole sentence besides the last word, further called target word) of those sentences with the first word that comes to mind (i.e., a sentence closing task). The average cloze probability of those sentences was 66.99% (*SEM* = 1.65). After that, four sentence groups were created by crossing factors of word association and sentence congruity (**Table 2**, note also the abbreviations per sentence group for quick referencing). Half of the original 280 sentences were kept congruent and the other half changed into incongruent ones. Incongruent sentences were composed by replacing the target word of a congruent sentence by one that does not semantically match the context of the sentence and renders it meaningless. In approximately half of both the congruent and incongruent sentences, the association between the target word (which was always a noun) and the closest open class word, i.e., a noun, verb, adverb, or adjective (i.e., the prime word), was present; in the other half, the association was absent. Three Dutch-speaking colleagues, who were blind to the sentence group, independently checked the meaningfulness of the congruent and incongruent sentences.

**Table 2:** Exemplar sentences in Dutch and their translations into English (for illustration purposes only).

Sentence group	Example sentence	Sentence translations
Congruent – associated (cong_HA)	Ze stak brandhout^†^ in de kachel^∗^	*She puts firewood^†^ into the stove^∗^*
Congruent – unassociated (cong_LA)	Met mijn familie heb ik weinig^†^ contact^∗^	*With my family I have little^†^ contact^∗^*
Incongruent – associated (incong_HA)	De operatietafel was bevlekt met etter^†^ en wonde^∗^	*Operational table was covered with pus^†^ and wound^∗^*
Incongruent – unassociated (incong_LA)	De leraar schreef zijn naam^†^ op het meer^∗^	*The teacher wrote her name^†^ on the lake^∗^*


*^†^Prime word; ^∗^Target word.*

The lexical characteristics of the target words were balanced across sentence groups in such a way that a repeated measure ANOVA did not show any significant difference between word frequencies [*F*(3,275) = 0.27, *p* = 0.85], checked with the SUBTLEX Dutch word frequency database (Keuleers et al., 2010), and both orthographic neighborhood (OTAN) [*F*(3,275) = 0.39, *p* = 0.76] and word length [*F*(3,275) = 0.82, *p* = 0.48] checked with the CLEARPOND non-commercial software (Marian et al., 2012).

Word association strength values between prime/target word-pairs in both congruent and incongruent sentences with associations present (cong_HA and incong_HA groups in **Table 2**) were taken from the word-association database of Flemish-Dutch word-pairs (De Deyne and Storms, 2008). This database was obtained by asking a large population of Flemish-Dutch subjects to list the three words that first come to mind when seeing a prime word (free association task). Association strength (AsSt) values of a given prime-target word pair is then expressed by the ratio between the number of subjects that replied with target word (NT arg et), in response to the prime, and the number of subjects (NTotal) presented with the prime: AsSt = NT arg et/NTotal.

In our case, the average association strength (with SEM in brackets) for the cong_HA group was 0.026 (0.0089) and incong_HA group was 0.1537 (0.0148). The Student’s *t*-test showed a statistically significant difference between these values (*p* < < 0.0001).

### Experimental Procedure

The experiment was conducted in a sound-attenuated, dimly lit room in the hospital (in case of patients) or experimental room in our laboratory (in case of healthy subjects). For the patients, the experiment consisted of two parts: the pencil and paper based Dutch version of the AAT (conducted by speech and language therapists MDL and GV, co-authors) and the computerized sentence comprehension test (on semantics) with simultaneous EEG recording (conducted by EK, first author). The order of the tests was counterbalanced across subjects, so that half of the patients did the AAT test first and the other half the computerized test. Patients also had a 30 min break between two tests to ensure that their performance would not be affected by fatigue.

For the computerized test, subjects were seated in a chair at a distance of approximately 70 cm from the LCD screen. Prior to the actual sentence comprehension test, for all subjects, an electro-oculogram (EOG) was recorded to clean EEG recordings from eye movements and blinks using the Revised Artifact-Aligned Averaging (RAAA) procedure described in Croft and Barry (2000). When recording eye movements, a white circle on a black background was moving vertically and horizontally starting from the center of the screen. Subjects were instructed to follow the circle with their eyes, without moving their head, and to refrain from blinking. When recording eye blinks, the same circle was coming down from the upper edge of the screen and was hitting a horizontal line that divided the screen. Subjects were instructed to focus on the center of the screen and to blink every time the circle hits the horizontal line. They were specifically cautioned not to track the circle. The following sentence comprehension test was split into six short blocks and subjects could take a short break every 5–7 min.

During the experiment, sentences were presented on the LCD screen, one word at a time, using white letters on a black background. Each word was presented during 500 ms with a jittered inter-stimulus interval (ISI) of approximately 300 ms on average (range of 200–500 ms). The ISI had a small jitter (±150 ms) in order to prevent an overlap of the ERPs generated in response to the previous words and to the target word (the latter is of our interest). At the end of each sentence, a blank screen appeared for 700 ms, which was followed by a question mark with two options [boxes with the words ‘Goed’ (correct) and ‘Fout’ (false)]. As soon as the subject saw the question mark, he/she should indicate whether the presented sentence was meaningful or meaningless by pressing the left or right mouse button, respectively (semantic anomaly judgment task). An explicit response was asked from the subject to keep him/her attentive and to ensure that each word was thoroughly processed. The button press response was delayed to avoid interference of the N400 with response related potentials (Van Vliet et al., 2014). We also used the button press responses to compare the behavioral data with the electrophysiological recordings. For both control groups the hand for the button press was counterbalanced across subjects.

The stimuli were presented using Matlab’s Psychophysics Toolbox (Brainard, 1997).

### EEG Acquisition

The EEG signal was acquired using 32 active Ag/AgCl electrodes mounted in a cap placed on the subject’s head according to the international extended 10–20 system. Additionally, six external electrodes were placed: two on the right and left mastoids for offline re-referencing of the EEG signal and one above and below the left eye, as well as one at the external canthus of each eye, to record vertical and horizontal eye movements, respectively. Conductive gel was applied to the surfaces of the external electrodes and into the halls of the electrode cap in order to improve conductance between those electrodes and the subject’s skin. The signal was acquired continuously and amplified with a BIOSEMI Active II System (BIOSEMI, Amsterdam, The Netherlands) with a built-in fifth order 0.16–100 Hz pass band filter and at a 2048 Hz sampling rate. The signal was down-sampled online from 2048 to 256 Hz. The signal quality was constantly inspected during the recordings.

For healthy controls, the whole experiment, including placing the electrodes, took around 1.5 h. For patients, with additional AAT testing and a 30 min break between two sessions, the whole experiment took around 2.5 h.

### Data Analysis

The recorded signal from each electrode was re-referenced offline from BIOSEMI’s common mode reference (CMR) to an averaged mastoid reference and filtered twice, using a fourth order Butterworth filter, a low-pass filtering with cutoff frequency of 15 Hz, and then a high-pass filtering with a cutoff frequency of 0.5 Hz. The correction of eye movement- and blink artifacts was conducted using the RAAA EOG correction method described in Croft and Barry (2000) using the external electrode recordings. Based on the calibration data, this algorithm calculates a coefficient **b**_{i,j}_ that describes the influence of EOG channel i on EEG channel j. Correction of the EEG is then done by subtracting the correct proportion of each EOG channel from each EEG channel. A description of the RAAA algorithm can be found in Appendix A of Croft and Barry (2000). After correcting on eye movement and eye blink artifacts, the signal was cut into epochs starting from 200 ms prior to the onset of the target word till 1000 ms post-onset. In order to further clean the data from remaining eye movement- and blink artifacts, as well as artifacts caused by muscle contraction or bad skin conductance, trials with amplitude larger than ±50 μV on any of the electrodes were rejected. Afterward, baseline correction was applied using the average signal in the 200 ms interval prior to the onset of the target word. For the trials that were kept (see above), amplitudes of the N400 and P600 responses were calculated as the average EEG amplitude in a time-interval, the beginning and duration of which varied across subject groups (young healthy subjects, older healthy subjects, and patients) depending on the latency and duration of the N400 potential. The time-interval of the P600 immediately followed that of the N400 and for all subjects chosen to end on 900 ms after presentation of the target word. The latter was motivated upon visual inspection of the epochs.

Prior to calculating the latency of the N400 potential for each subject group, we visually inspected a single subject’s average ERP as well as the grand average ERP across subjects of each subject group per electrode. Then, we calculated the exact latency for electrode Cz based on the method described in Luck (2005). According to this method, we performed an ANOVA with sentence group (four levels) as a fixed factor^2^ for each time point of the average ERPs of all subjects within each subject group, starting from 250 ms post-stimulus onset and detect the first instance where the effect of sentence group was significant. If the significance of this effect was consistent over a 50 ms time interval (13 consecutively significant ANOVAs), we considered this instance as the onset latency of the N400 potential.

The offset and, therefore, duration of the N400 potential for each subject group was estimated by visually inspecting a single subject’s average ERP, as well as the grand average ERP across subjects of each subject group.

A subsample of 28 electrodes (all, besides the most frontal ones: Fp1, Fp2, AF3, and AF4) from 32 used for recording was chosen to investigate the effect of laterality only. The choice was made based on the known spatial distribution of the N400 potential (Kutas and Federmeier, 2011). All other analyses were conducted on all 32 electrodes.

Data analysis was done in Matlab using the BIOSIG bio-signal processing toolbox (Vidaurre et al., 2011), scalp plots were plotted using EEGLAB’s eeg_topoplot function (Delorme and Makeig, 2004).

### Statistical Analysis

For the behavioral data, a mixed design analysis of variance (ANOVA) was applied (Verbeke and Molenberghs, 2000) with sentence congruity (Con., two levels: congruent and incongruent) and association (AS, two levels: with and without association) as intra-group- and subject group (SubG, three levels) as inter-group independent fixed variables and their interactions, and performance accuracy as dependent variable. For the EEG analysis, to keep the model simple, we separately compared each pair of subject groups in order to assess which ones were different. At the end, we had three SubG × Con × AS models with a 2 × 2 × 2 design: comparisons between young and older healthy controls, young controls and patients and older controls and patients. When evaluating each subject group separately, we studied the effects of Con, AS, and their interaction, on the N400 and P600 amplitudes within each subject group following a 2 × 2 structure. Additionally, we included subject as random effect in order to correct for associations within each subject. For further multiple comparisons, the Student’s *t*-test was used with the Satterthwaite approximation for degrees of freedom, where appropriate, and Benjamini-Hochberg FDR correction for *p*-values (Benjamini and Hochberg, 1995). A significance level of 5% was kept across the entire analysis.

In order to assess the effect of N400 scalp distribution, depending on subject group, we developed the statistical model to compare pairs of subject groups with the following fixed effects: left–right (three levels, left, medial, right), frontal–occipital (three levels, frontal, central, parieto-occipital) and subject group (SubG, three comparisons with two levels: young versus older control, young control versus patients, older control versus patients). As dependent variable, the mean N400 amplitude from the selected subsample of 28 electrodes was considered. The N400 amplitude for each trial was calculated by taking the mean ERP amplitude between onset – defined for each subject group using Luck’s method (see Data Analysis) – and offset defined by visual inspection (again, see Data Analysis).

For the patient group, the Pearson’s correlation was performed between the results of each subcomponent of the AAT test and the average amplitude of the N400 effect: the difference between N400 amplitude in response to each of the sentence groups (SG) and N400 amplitude in response to the incong_LA group (N400_SG_ – N400_incong_LA_), for each subject group, as well as performance accuracy (behavioral data from semantic anomaly judgment task).

## Results

### Behavioral Data

Mixed design ANOVA with sentence congruity (Con., two levels), association (AS, two levels), subject group (SubG, three levels) and their interactions as independent factors showed a significant effects of Con [*F*(1,178) = 8.16, *p* = 0.0048], AS [*F*(1,178) = 4.77, *p* = 0.03], SubG [*F*(2,178) = 23.47, *p* < < 0.00001] and some of their interactions: Con × AS (*p* = 0.0013), and Con × SubG (*p* = 0.017). In general, patients performed worse compared to both control groups (*p* < 0.0005 in both comparisons): average hit-rate (with SEM in brackets) for patients was 0.876 (0.0372), for young healthy participants 0.964 (0.0075) and older healthy participants 0.963 (0.0103). The performance accuracy for each sentence group in each subject group is presented in **Supplementary Table S1**.

**FIGURE 1:**
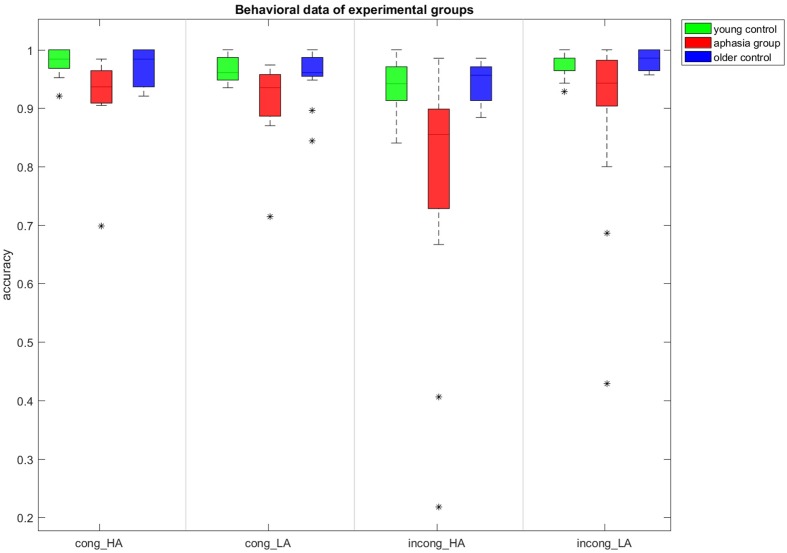
**Average performance for each subject group in each sentence group.** The error bars represent the minimum and maximum performance without outliers (asterisks). The horizontal line in the middle of the bar represents the median and the lower and upper limits of the boxes indicate first and third quartiles respectively.

*Post hoc* pairwise comparison showed no statistical difference between performance accuracy in young and older healthy controls, for any of the sentence groups. A Student’s *t*-test showed a statistically significant difference between patient group and young control group (**Figure 1**) for all sentence groups (*p* < 0.05) except for the incong_LA group where the difference was non-significant (*p* = 0.054). When comparing each sentence group between older controls and patients, the differences between performances on cong_HA (*p* = 0.036) and incong_HA (*p* = 0.011) were statistically significant. The differences between other two groups cong_LA (*p* = 0.057) and incong_LA (*p* = 0.055) were not statistically significant.

Within each subject group, a *post hoc* pairwise comparison using Student’s *t*-test showed that meaningless sentences with association between words were significantly less accurately responded compared to other sentence groups for healthy young controls (*p* < 0.01 for all comparisons). Unlike this, for the older control group, the only significant difference between sentence groups was a difference in performance between the incong_HA and incong_LA sentence groups (*p* = 0.0043). For the aphasia group, the performance on incong_HA had a statistically significant lower accuracy compared to both cong_HA (*p* = 0.018) and cong_LA (*p* = 0.0265), however the performance on incong_HA in patients was statistically not different from incong_LA (*p* = 0.09).

For the patient group, a Pearson’s correlation ran between the results of each subcomponent of the AAT test (Token Test, repetition, writing, naming, and comprehension) and the accuracy levels of each sentence group showed a significant correlation for several cases (**Table 3**).

**Table 3:** Correlation table between performance of patients on AAT subtests and their behavioral results for each sentence group.

AAT subtest results	Sentence group			
	
	cong_HA	cong_LA	incong_HA	incong_LA
Token test	0.548	0.641^∗^	0.378	0.291
Repetition	0.1385	0.215	0.341	0.117
Writing	0.663^∗^	0.413	0.424	0.246
Naming	-0.115	-0.167	0.484	0.4035
Comprehension	0.106	0.353	0.758^∗∗∗^	0.743^∗∗∗^
*p* = 0.05	*^∗^p* < 0.05	*^∗∗^p* < 0.01	*^∗∗∗^p* < 0.005	


*Values represent correlation strength (Rho), asterisks - the significance levels of the correlations.*

### ERP Data

Two patients (E. J. and P. I.) were excluded from ERP analysis because of excessive artifacts (after cleaning the data only eight trials were kept in E. J. and three trials in P. I.), hence, we report on ERPs from the remaining 13 patients. From the remaining subjects, in total 3% of the trials from the young control group, 30% from the older controls and 10% from the remaining 13 patients group were considered as artifact–contaminated (exceeding the ±50 μV threshold on any electrode) and were removed from further analysis.

#### Latency

The average latency onset of the N400 potential for healthy young controls, using Luck’s method described above, was 274.2 (*SEM* = 20.04). For this group, the time range of the N400 potential was from 274.2 till 500 ms. For the older control group, the latency was 300 ms (*SEM* = 14.47) and the range accordingly from 300 till 500 ms. For the patient group, the N400 latency onset was 368.1 ms (*SEM* = 26.7) whence, for this group, the time-range of N400 potential was chosen as 368.1–579.9 ms. Therefore, from now on, when referring to the N400 potential or N400 effect we will rely on the average EEG amplitude given the time range for each of the subject groups. For the P600 potential, the latencies were chosen as 500 ms for healthy individuals and 580 ms for aphasia patients.

We also performed a detailed temporal analysis of the N400 potential and found evidence for differences in N400 shape between healthy controls and patients (see Supplementary Material, “Evaluation of 50 ms time windows to compare N400 onset latencies” section) that could explain the observed differences in N400 latency between those groups.

#### Amplitude

**FIGURE 2:**
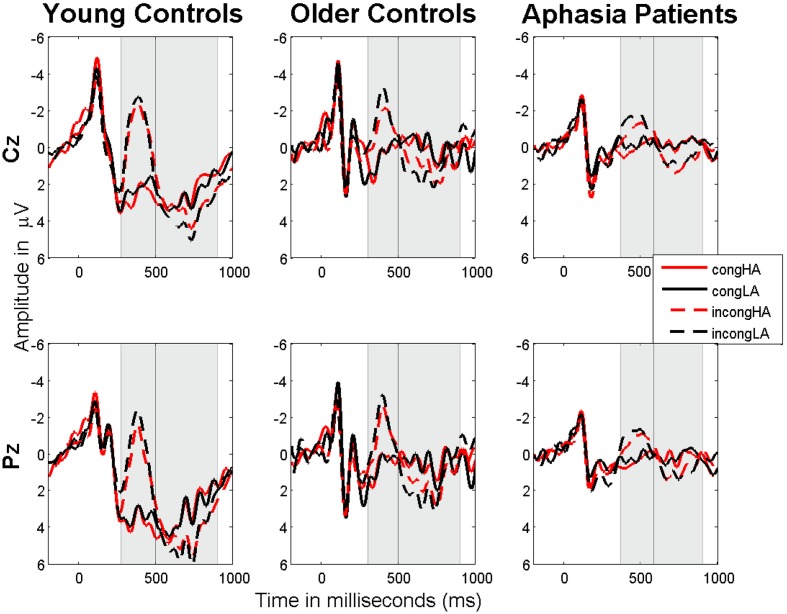
**N400 and P600 potentials on central electrodes Cz and Pz for each subject group (young healthy controls, older healthy controls, and aphasia patients).** The gray shaded areas represent the statistically significant differences between sentence groups. Negative voltages are plotted upward. The decrease in N400 amplitude in older control subjects and patients compared to the young control group and the increase in N400 onset latency in the patients compared to both control groups are observed.

As the number of trials per subject for each sentence group was not always the same (mainly following artifact rejection), the ERP effect for both N400 and P600 for each sentence group was assessed by taking the grand average effect for that particular sentence group over each subject’s N400 effects within each subject group according to the following formula:

$${GrandAve}_{N400effect}=\frac{\Sigma_{i=1}^{N}{N400effect}_{i}}{N},$$

where N is the number of subjects, _N400effect_i__ the N400 effect for subject *i*, calculated with the following formula: (_^ave^N400SG-^ave^N400incongLA_), where ^ave^N400SG is the average N400 amplitude in response to the given sentence group and _^ave^N400incongLA_ is the average N400 amplitude in response to the incong_LA sentence group for the individual subject. **Table 4** lists the mean and SEM of the N400 and P600 effect sizes on electrode Cz for each sentence group and subject group. Effect size for P600 is calculated the same way as for N400, but considering the appropriate time window. **Figure 2** represents the ERP plots for the central electrodes (Cz, Pz) of each subject group (young healthy controls, older healthy controls, and aphasia patients).

**Table 4:** Mean and SEM (in brackets) of N400 and P600 effect sizes for each sentence group for each subject group on electrode Cz.

Subjects group	N400			P600		
		
	cong_HA	cong_LA	incong_HA	cong_HA	cong_LA	incong_HA
Young healthy	3.57 (1.08)	3.16 (0.99)	0.204 (0.68)	-1.12 (0.76)	-1.034 (0.95)	-0.49 (0.72)
Older healthy	1.76 (1.86)	1.89 (1.4)	0.335 (1.03)	-1.9 (1.61)	-1.94 (1.61)	-0.29 (0.72)
Aphasia	1.31 (0.81)	1.14 (0.69)	0.41 (0.37)	-0.67 (0.42)	-0.76 (0.49)	0.33 (0.61)


When applying a mixed effect model ANOVA on each pair of subject groups with the inclusion of subject group (SubG: young versus older healthy, young healthy versus patient, and older healthy versus patient) as inter-group- and sentence congruity (Con., two levels), and association (AS, two levels) as intra-group, and their interactions (2 × 2 × 2 design) as independent variables and ERP average amplitudes (N400 and P600) in response to each sentence group of each subject group on electrode Cz as dependent variable, the following results were observed (**Table 5**). When comparing the young healthy group with the other two groups, effects of SubG, Con and their interaction (SubG × Con) were significant for both time windows (N400 and P600) for both comparisons, except for the effect of SubG × Con interaction on P600 (both comparisons). When comparing the older healthy group with the aphasia group the significant effect of SubG was observed only for the P600 potential, while the effect of Con was significant for both potentials. The effects of AS (*p* = 0.037), as well as SubG × Con interaction, were significant only for the N400 potential (**Table 5**).

**Table 5:** *F*-values for the effects of SubG, SG and SubG × SG interaction on the amplitudes of N400 and P600 potentials.

	N400			P600		
		
	YC versus OC	YC versus P	OC versus P	YC versus OC	YC versus P	OC versus P
SubG	***F* = 26.06^∗∗∗^**	***F* = 58.11^∗∗∗^**	*F* = 2	***F* = 173.86^∗∗∗^**	***F* = 343.96^∗∗∗^**	***F* = 7.34^∗∗^**
AS	*F* = 1.65	*F* = 2.88	***F* = 4.37^∗^**	*F* = 1.16	*F* = 0.45	*F* = 0.05
Con.	***F* = 110.12^∗∗∗^**	***F* = 128.89^∗∗∗^**	***F* = 94.39^∗∗∗^**	***F* = 22.11^∗∗∗^**	***F* = 30.01^∗∗∗^**	***F* = 58.6^∗∗∗^**
Con × AS	*F* = 0.12	*F* < < 1	*F* = 1.24	*F* = 0.32	*F* = 0.13	*F* = 0.04
SubG × Con	***F* = 8.14^∗∗^**	***F* = 32.35^∗∗∗^**	***F* = 6.64^∗^**	*F* = 0.03	*F* = 0.04	*F* = 0.32
SubG × AS	*F* < < 1	*F* < < 1	*F* = 0.01	*F* = 0.16	*F* = 1.41	*F* = 0.83
SubG × Con × AS	*F* = 0.45	*F* = 0.19	*F* = 0.27	*F* = 0.16	*F* = 0.74	*F* = 0.23
***Degrees of freedom (DF):***		*YC* versus *OC*		1, 6692		
		*YC* versus *P*		1, 8362		
		*OC* versus *P*		1, 5172		


*The significant values are presented in bold. Significance levels are marked with asterisks and are represented with the following p–values: *^∗^p* < 0.05, *^∗∗^p* < 0.01, and *^∗∗∗^p* < 0.005. Coding of the subject groups is as follows: YC, young control; OC, older control; P, patients.*

We further studied the effects of congruity (Con.), lexical association (AS) and their interaction on the N400 and P600 amplitudes within each subject group separately. The 2 × 2 unstructured linear mixed effects model with subject as random effect within each subject group, applied to the mean amplitudes of N400 and P600 for the corresponding time-ranges, showed a significant effect of Con. on both N400 and P600 amplitudes of each subject group. For electrode Cz, the *F* and *p* values were as follows: young controls N400 *F*(1,5015) = 69.98 (*p* < < 0.0001), P600 *F*(1,5015) = 12.03 (*p* = 0.002), older controls N400 *F*(1,1873) = 10.46 (*p* = 0.0071), P600 *F*(1,1873) = 16.16 (*p* = 0.0023), and aphasia patients N400 *F*(1,3221) = 10.32 (*p* = 0.0068), P600 *F*(1,3221) = 17.36 (*p* = 0.0009). Neither the effect of AS nor Con. × AS interaction were significant in any of the subject groups for any of the mentioned potentials (for all groups *p* > > 0.05). A *post hoc* pairwise comparison (Student’s *t*-test) of N400 and P600 amplitudes across sentence groups within each subject group (*t*- and *p*-values listed in **Table 6**) revealed a similar picture across the subject groups. As we can see from the **Table 6** and **Figure 2**, the N400 amplitudes in response to both congruent sentence groups (cong_HA and cong_LA) were significantly smaller compared to the ones in response to both incongruent groups (incong_HA and incong_LA). No significant difference was detected between N400 amplitudes in response to the cong_HA and cong_LA, as well as incong_HA and incong_LA sentence groups. Similarly, both incongruent sentence groups evoked equally stronger positivities in the later time window (P600) compared to the congruent sentences. Note, that for older controls the difference between cong_HA and incong_HA for P600 was no longer significant after correcting for multiple comparisons.

**Table 6:** *t*- values for pairwise comparison (Student’s *t*-test) of N400 and P600 amplitudes between sentence groups within each subject group with FDR correction for multiple comparisons.

*Comparison*	Young Controls		Older Controls		Aphasia Patients	
			
	*N400*	*P600*	*N400*	*P600*	*N400*	*P600*
*cong_HA* versus *cong_LA*	<<1 (2363)	<1 (2527)	<<1 (968)	<1 (881)	<<1 (1641)	<1 (1650)
*cong_HA* versus *incong_HA*	7.94^∗∗∗^ (2315)	4.9^∗∗∗^ (2395)	4.46^∗∗∗^ (877)	2.08 (899)	3.85^∗∗∗^ (1535)	4.18^∗∗∗^ (1537)
*cong_HA vs incong_LA*	8.5^∗∗∗^ (2356)	5.85^∗∗∗^ (2459)	5.998^∗∗∗^ (910)	2.65^∗^ (910)	5.69^∗∗∗^ (1553)	3.62^∗∗∗^ (1554)
*cong_LA* versus *incong_HA*	7.64^∗∗∗^ (2632)	4.08^∗∗∗^ (2632)	4.56^∗∗∗^ (991)	3.12^∗∗^ (991)	2.86^∗∗^ (1715)	4.59^∗∗∗^ (1715)
*cong_LA* versus *incong_LA*	8.27^∗∗∗^ (2696)	5.05^∗∗∗^ (2662)	6.18^∗∗∗^ (1002)	3.7^∗∗∗^ (953)	4.65^∗∗∗^ (1729)	4^∗∗∗^ (1732)
*incong_HA* versus *Incong_LA*	<<1 (2564)	<1 (2564)	<<1 (933)	<1 (933)	<<1 (1619)	<1 (1619)


*Significance level is presented with asterisks. The coding of significance level is as for the previous tables. The degrees of freedom (DF) are presented in brackets next to the *t*-values.*

As the N400 is the main focus of the current article, and since the P600 results are similar to the ones for N400, the results for the following analyses are only shown for the N400.

#### Correlation Analysis

For two of the 13 artifact-free patient recordings included in the ERP study, there were no AAT test results (**Table 1**); hence, we excluded them from the correlation analysis.

**FIGURE 3:**
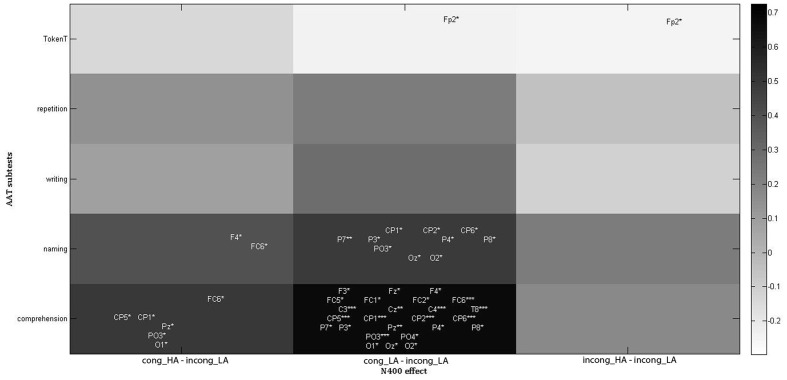
**Correlations between patient performance on AAT subtests and N400 effect size for each sentence group.** The color bar represents correlation strength. The locations of electrode names show the approximate locations of the electrodes. Asterisks indicate the level of significance: ^∗^*p* < 0.05, ^∗∗^*p* < 0.01, ^∗∗∗^*p* < 0.005. For Rho values of significant correlations on each electrode position see **Supplementary Table S2**.

The Pearson’s correlation between the outcome of the AAT subtests and the N400 effect for each sentence group (**Figure 3**) showed a significant positive correlation between comprehension subtest and N400 effect in response to the cong_LA sentence group on a number of channels (see **Figure 3**). For example, for electrode Pz, this correlation was ρ = 0.772, *p* = 0.0054, for electrode C4 – ρ = 0.828, *p* = 0.0017. Additionally, the outcome of the comprehension subtest positively correlated with N400 effect in response to cong_HA on a number of electrodes (**Figure 3**). Similarly, the naming subtest score correlated positively with N400 effect in response to cong_LA and cong_HA (the last one to a lesser extent). Finally, on electrode Fp2, the Token Test score correlated negatively with N400 effect in response to both cong_LA and incong_HA groups.

#### Laterality Effect

**FIGURE 4:**
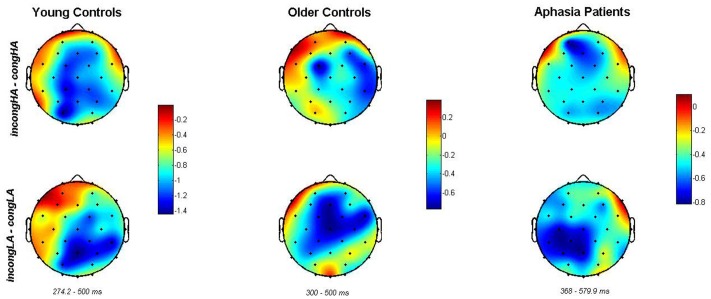
**Spatial distribution of N400 difference between congruent and incongruent sentences (top row: incong_HA – cong_HA, bottom row: incong_LA – cong_LA) across different subject groups (column-wise) given the time-range for each group.** Color bars to the right of each pair of scalp plots in the same column refer to the amplitude difference between the mentioned sentence groups of the same subject group. The change in spatial distribution of negative activity (N400) across subject groups is presented.

The selected 28 electrodes were divided into 9 groups: left-frontal (F3, F7, FC5), medial-frontal (FC1, Fz, FC2), right-frontal (F4, F8, FC6), left-central (T7, C3, CP5), central (CP1, Cz, CP2), right-central (C4, CP6, T8), left parieto-occipital (P7, P3, PO3), medial parieto-occipital (Pz, O1, Oz, O2), and right parieto-occipital (P4, P8, PO4). In order to investigate how the N400 effect changes spatially (**Figure 4**), depending on subject group (the effects of aging and brain impairment causing aphasia), we applied mixed effect ANOVA on each pair of subject groups (SubG: young versus older controls, young controls versus patients and older controls versus patients). Hence, we had three 3 × 3 × 2 models (left–right × frontal-occipital × SubG). When comparing young and older controls, the effects of left–right (*p* = 0.001), frontal-occipital (*p* < < 0.001), and subject group (*p* < < 0.001) were significant. Similarly, the interactions between the following factors: frontal-occipital × left–right (*p* < < 0.001), frontal-occipital × SubG (*p* < < 0.001) and left–right × SubG (*p* < < 0.001), as well as the three-way interaction left–right × frontal-occipital × SubG (*p* < < 0.001) were significant.

The results of the comparison between young controls and patients showed significant effects of all included factors: frontal-occipital, left–right and SubG (*p* < < 0.001 in all cases), as well as their interactions: frontal-occipital × left–right (*p* < < 0.001), frontal-occipital × SubG (*p* < < 0.001) and SubG × left–right (*p* = 0.0123). The effect of the three-way interaction was also significant (*p* < < 0.001).

Similarly, when comparing older healthy control group with patients, effects of all factors and their interactions were significant (in all cases *p* < < 0.0001).

## Discussion

We investigated the contribution of word-association in sentence processing in patients suffering from aphasia but with relatively spared comprehension. Our hypothesis was that word-association can have a significant role in sentence processing in aphasia patients and that the N400 and P600 ERPs in response to sentences with associations would be different from the ones in response to sentences without associations. To test our hypothesis, we recorded EEG in aphasia patients, young and older healthy controls in response to semantically congruent and incongruent sentences, equally divided into sentences with and without associations between prime and target words. Simultaneously, we recorded behavioral responses from our subjects and obtained the AAT results from aphasia patients. The N400 ERP results for our patients showed that here, as in healthy controls, sentence-level information overrides word-level information, which was shown by a significant effect of congruity and further pairwise comparison: in all three subject groups, the N400 amplitudes were smaller in response to congruent sentences compared to incongruent ones. On the other hand, unlike healthy young controls, when comparing older controls with aphasia patients, we observed a significant effect of AS, suggesting that word association plays some role in sentence processing for these two groups, which could also be age-related. But note that this is a very small effect as it shows up only when comparing older controls and patients but not when investigating each subject group separately. The presence of an association effect in our patient group can be further strengthened by another observation of the current study: a positive correlation between the outcome of the comprehension subtest of AAT and the N400 effect in response to cong_LA sentences for the centro-parietal electrodes (representative for N400 responses). This suggests that patients with more pronounced comprehension deficit will have a smaller N400 effect in response to cong_LA sentences, therefore a larger N400 potential in response to this sentence group and that these sentences could be more frequently perceived as incongruent by these patients. This observation is similar to Coulson et al.’s (2005) as they observed a small effect of lexical association in congruent sentences when presenting them in the left visual hemifield (right hemisphere). It also suggests that patients with more pronounced comprehension deficit (even if this deficit was still mild in those patients, see **Table 1**) will pay more attention to associations between words in congruent sentences, which facilitates their processing, compared to patients with better preserved comprehension. It is worth mentioning that, as shown by the results of behavioral data, the patients’ performance on these sentences as a whole was not worse compared to the other congruent sentence group (cong_HA). As all our patients had relatively spared (or recovered) comprehension (**Table 1**), the observed correlation between mild changes in the outcome of the comprehension subtest of AAT and the ERP result can be a consequence of the explicit task used. Indeed, Kojima and Kaga (2003) showed that mild lexical-semantic impairments can be better detected with the N400 potential when the task is explicit rather than implicit. A similar correlation was observed between N400 effect of cong_LA group and naming subtest scores (again with centro-parietal spatial distribution), which might assume that the N400 potential serves as a predicting factor also for the patients’ naming abilities.

Unlike Coulson et al. (2005), who observed an effect of word-association, when presenting these associations to only one visual hemifield, the effect of association (AS) was not significant in our aphasia patient group when tested separately (note that our patients also had a left hemisphere impairment). A possible explanation is that, after the left hemisphere incident, the right hemisphere gets involved into processing of congruity, hence, the facilitatory effect this hemisphere had for word association processing in healthy subjects (like in case of Coulson et al., 2005), gained less significance after recovery. This can be considered as a compensatory mechanism, which is also supported by the observed shift of N400 spatial distribution in aphasia patients compared to older healthy controls (**Figure 4**). As all our patients underwent rehabilitation, basing ourselves on the results of our laterality study (**Figure 4**, different spatial distributions for healthy older controls and patients), we can assume that the generator(s) of the N400 ERP might include additional brain areas after rehabilitation, as a sign of compensation. This is also in accordance with the literature where the spatial distribution of the N400 potential shifted after intensive speech language therapy (Wilson et al., 2012). On the other hand, when comparing older controls with aphasia patients, we did observe a significant effect of AS, suggesting that in both healthy older and patient groups a mild effect of association might be present. Additionally, the significant correlation between comprehension level and N400 effect of cong_LA sentences suggests that patients with more impaired comprehension might still rely on word-association when processing meaningful sentences. This is a subject of further study.

Our study also confirms previous reports (Swaab et al., 1997; Kawohl et al., 2010) that lexico-semantic information in patients with aphasia is not lost, but rather the integration of this information into the working context is delayed as we observed a delayed N400 response in this subject group compared to both healthy control groups. Similar to some other studies, we found no significant increase in latency in our older control group (Faustmann et al., 2007; Tsolaki et al., 2015). Instead, we observed a reduction in N400 amplitude, which was also previously demonstrated (Kutas and Iragui, 1998; Federmeier and Kutas, 2005) (though note that they also observed an increased latency). The absence of a significant effect of subject group (SubG) on N400 amplitude, when comparing older control and patient groups, supports the notion that the N400 potential of aphasia patients with mild comprehension deficit is comparable to that of healthy age-matched controls in terms of amplitude (Hagoort et al., 1996; Swaab et al., 1997). On the other hand, the increased N400 latency in those patients indicates a delayed semantic integration (though still normal given the N400 amplitude) of the word into working context of the sentence compared to age matched (older) controls. This is in accordance with several studies that reported on the N400 to reflect semantic integration in sentence or discourse context (Curran et al., 1993; van Berkum et al., 1999). A similar observation was also reported by Kawohl et al. (2010).

As to the P600 potential, we observed this potential together with N400 for all sentences and subject groups. In the study of van de Meerendonk et al. (2010), it was shown that strong semantic violation, e.g., “The eye consisting of among other things a pupil, iris and *sticker*….,” can evoke both N400 and P600, whereas mild semantic violation, e.g., “The eye consisting of among other things a pupil, iris and *eyebrow*….,” evokes only N400. Therefore, as we observed an equally large P600 for both incongruent associated and unassociated sentences, we can assume that their semantic incongruity was equally strong. We observed no effect of association on P600 for any of the comparisons. This confirms the previous statement that sentences with and without associations with similar incongruity levels are processed in a similar manner also in terms of further re-analysis. However, unlike N400, there was a significant effect of SubG on P600 amplitudes when comparing older controls and aphasia patients, with a larger and better defined P600 for the aphasia patients (**Figure 2**). This indicates that, in patients with aphasia, the later and probably more consciously evoked P600 component is more involved in the processing of semantic violation compared to age matched healthy individuals.

Another point worth mentioning is the difference between behavioral and EEG results in our study. The behavioral results showed that patients (but also young controls) performed worse on incong_HA sentences compared to congruent groups of sentences. Additionally, the comprehension subtest score of AAT in the patient group was positively correlated with the accuracy levels of both incongruent sentence groups. Hence, we can assume that patients with more severe comprehension deficit were by default considering sentences as congruent. However, our ERP study did not reveal any significant difference between processing incong_HA and incong_LA sentence groups in any of the subject groups. This might suggest the benefit of the N400 potential when investigating separate components of language processing independently from behavioral response.

There is one more general point to address: whether it is possible that long distance associations define the congruity of our sentences and whether it would be possible to have congruent sentences without associations in general. To address this question, we first remind the two main hypotheses about sentence processing in the brain (Lau et al., 2008), especially in terms of N400 generation. According to the integration hypothesis (Kutas and Hillyard, 1980; Borovsky et al., 2012), when a sentence is read, every upcoming word enters a “buffer” of working memory and tries to be integrated. If integration is not possible, this sentence is perceived as incongruent. According to the prediction hypothesis (Lau et al., 2013), the words in the sentence evoke predictions about the upcoming word and when violated, the sentence is perceived as incongruent. In both hypotheses, it can be assumed that the context is developed from both syntactically correct formulations: words in specific categories and forms as well as appropriate semantic formulations: words that should, to some extent, have thematic and/or semantic/associative relations in the sentence. This would especially be true for sentences with high contextual constraint (CP > 95%). However, our stimuli contained a significant number of sentences with low to middle constraint (CP ranges 24.32–97.44), which were equally distributed across sentence groups. Therefore, the final words of originally congruent sentences (all sentences in the stimulus list) did not always have a high expectation. This suggests that the associations in those sentences were not always very strong and whence (especially the long range ones) not necessarily defining the congruity of the sentence.

On the other hand, as the previous words cumulate and build up the context, they should to some extent be connected (even weakly associated – semantically or thematically) to the target word in congruent sentences (both associated and unassociated). Yet, both our meaningless and meaningful sentences presented no effect of association. Additionally, our meaningful sentences differed from each other only based on one association between prime and target words (as according to the above mentioned logic, the long range associations would be present in both sentence groups), hence, keeping other factors between previous context and target words constant. Therefore, as our manipulation was done based on the closest prime-target relations in both groups of meaningful sentences, which was controlled for the presence or absence of associations, we can safely assume that long distance associations should not influence our results.

Our study might also have some clinical implications. We started with the hypothesis that in aphasic patients, word association might play a more significant role in comprehending sentence level information compared to healthy controls. However, our results only partially confirmed our hypothesis: a small effect of AS was observed when only comparing older controls with aphasia patients. The reason for this might be the fact that in our study we tested mild to minimal comprehension deficit patients. However, the observed correlations indicate that for more severe comprehension deficit patients, word-level information could indeed play a more significant role in processing of sentence-level information in congruent context compared to the healthy individuals. Therefore, when developing rehabilitation strategies based on semantic-relatedness, it could be beneficial to consider both word- and sentence-level information. Indeed, although patients after rehabilitation (all our patients underwent rehabilitation) could adhere to the “meaning first” principle, as healthy subjects do, the ones with less improved comprehension could additionally adopt another, compensatory strategy to improve comprehension. One of those strategies could be relying on existing associations between words in the presented sentences. But note that this was the case when these two information types (word level and sentence level) did not contradict each other, i.e., these words were embedded in congruent sentences. As a recommendation for future study, one would need to test patients with more pronounced comprehension deficits in order to further unveil the value of word-level information in sentence processing.

## Conclusion

Aphasia patients with mild to minimal comprehension deficits process sentence- versus word-level information very much in the same way as healthy subjects: yielding to sentence level information in processing conflicting sentences (meaningless sentences with association between words). On the other hand, patients with more pronounced comprehension deficit, when processing meaningful sentences, could additionally rely on word-level information.

## Author Contributions

EK, AG, and MVH designed the study. MDL, GV, and AG recruited patients and older control subjects. MDL and GV conducted paper and pencil aphasia test on patients and analysis of data from aphasia test. EK conducted EEG study and EEG data analysis, as well as statistical analysis of the whole data. EK and MVH wrote the manuscript. All co-authors revised manuscript and had their contribution in creating the final version of manuscript.

## Conflict of Interest Statement

The authors declare that the research was conducted in the absence of any commercial or financial relationships that could be construed as a potential conflict of interest.

## Footnotes

1 From now on, the words “association” and “relatedness” will be used interchangeably.

2 Here, we used sentence group as a fixed factor instead of Con x AS design, as we only wanted to see when the effect starts to be significant, rather than to check for the difference between groups.

## References

[B1] Aerts A. (2014). *Neurophysiological and Clinical Investigation of Phonological Input Processing in Non-Brain Damaged Individuals and Patients with Aphasia.* Ph.D. dissertation, Ghent University, Ghent.

[B2] Basso A. Capitani E. Zanobio M. E. (1982). Pattern of recovery of oral and written expression and comprehension in aphasic patients. *Behav. Brain Res.* 6 115–128. 10.1016/0166-4328(82)90009-2 7138643

[B3] Benjamini Y. Hochberg Y. (1995). Controlling the false discovery rate: a practical and powerful approach to multiple testing. *J. R. Stat. Soc.* 57 289–300. 10.2307/2346101

[B4] Borovsky A. Elman J. L. Kutas M. (2012). Word meanings from a single exposure in context. *Lang. Learn. Dev.* 8 278–302. 10.1080/15475441.2011.614893.Once 23125559 PMC3484686

[B5] Brainard D. H. (1997). The psychophysics toolbox. *Spat. Vis.* 10 433–436. 10.1163/156856897X00357 9176952

[B6] Brouwer H. Hoeks J. C. J. (2013). A time and place for language comprehension: mapping the N400 and the P600 to a minimal cortical network. *Front. Hum. Neurosci.* 7:758 10.3389/fnhum.2013.00758 PMC3824103 24273505

[B7] Brown C. Hagoort P. (1993). The processing nature of the N400: evidence from masked priming. *J. Cogn. Neurosci.* 5 34–44. 10.1162/jocn.1993.5.1.34 23972118

[B8] Coulson S. Federmeier K. D. Van Petten C. Kutas M. (2005). Right hemisphere sensitivity to word- and sentence-level context: evidence from event-related brain potentials. *J. Exp. Psychol. Learn. Mem. Cogn.* 31 129–147. 10.1037/0278-7393.31.1.129 15641911

[B9] Croft R. J. Barry R. (2000). Removal of ocular artifact from EEG: a review. *Neurophysiol. Clin.* 30 5–19. 10.1016/S0987-7053(00)00055-1 10740792

[B10] Curran T. Tucker D. M. Kutas M. Posner M. I. (1993). Tomography of the N400: brain electrical activity reflecting semantic expectation. *Electroencephalogr. Clin. Neurophysiol.* 88 188–209. 10.1016/0168-5597(93)90004-9 7684968

[B11] Daltrozzo J. Wioland N. Kotchoubey B. (2012). The N400 and late positive complex (LPC) effects reflect controlled rather than automatic mechanisms of sentence processing. *Brain Sci.* 2 267–297. 10.3390/brainsci2030267 24961195 PMC4061799

[B12] Dambacher M. Kliegl R. Hofmann M. Jacobs A. M. (2006). Frequency and predictability effects on event-related potentials during reading. *Brain Res.* 1084 89–103. 10.1016/j.brainres.2006.02.010 16545344

[B13] D’Arcy R. C. Marchand Y. Eskes G. A. Harrison E. R. Phillips S. J. Major A. (2003). Electrophysiological assessment of language function following stroke. *Clin. Neurophysiol.* 114 662–672. 10.1016/S1388-2457(03)00007-5 12686275

[B14] De Deyne S. Storms G. (2008). Word associations: network and semantic properties. *Behav. Res. Methods* 40 213–231. 10.3758/BRM.40.1.213 18411545

[B15] Deacon D. Hewitt S. Yang C. M. Nagata M. (2000). Event-related potential indices of semantic priming using masked and unmasked words: evidence that the N400 does not reflect a post-lexical process. *Cogn. Brain Res.* 9 137–146. 10.1016/S0926-6410(99)00050-6 10729697

[B16] Delorme A. Makeig S. (2004). EEGLAB: an open source toolbox for analysis of single-trial EEG dynamics including independent component analysis. *J. Neurosci. Methods* 134 9–21. 10.1016/j.jneumeth.2003.10.009 15102499

[B17] Faustmann A. Murdoch B. E. Finnigan S. P. Copland D. A. (2007). Effects of advancing age on the processing of semantic anomalies in adults: evidence from event-related brain potentials. *Exp. Aging Res.* 33 439–460. 10.1080/03610730701525378 17886018

[B18] Federmeier K. D. Kutas M. (2005). Aging in context: age-related changes in context use during language comprehension. *Psychophysiology* 42 133–141. 10.1111/j.1469-8986.2005.00274.x 15787850

[B19] Freitas G. (2012). Aphasia and other language disorders. 30 41–45. 10.1159/000333402 22377860

[B20] Goodglass H. Kaplan E. (1983). *Boston Diagnostic Aphasia Examination.* Malvern, PA: Lea & Febinger.

[B21] Graetz P. De Bleser R. Willmes K. (1991). *Aachen Aphasia Test. Dutch version.* Lisse: Swets & Zeitlinger.

[B22] Grindrod C. M. Baum S. R. (2003). Sensitivity to local sentence context information in lexical ambiguity resolution: evidence from left- and right-hemisphere-damaged individuals. *Brain Lang.* 85 503–523. 10.1016/S0093-934X(03)00072-5 12744960

[B23] Hagoort P. (2003). Interplay between syntax and semantics during sentence comprehension?: erp effects of combining. *J. Cogn. Neurosci.* 15 883–899. 10.1162/089892903322370807 14511541

[B24] Hagoort P. Brown C. Swaab T. (1996). Lexical—semantic event–related potential effects in patients with left hemisphere lesions and aphasia, and patients with right hemisphere lesions without aphasia. *Brain* 119 627–649. 10.1093/brain/119.2.627 8800953

[B25] Hagoort P. Hald L. Bastiaansen M. Petersson K. M. (2004). Integration of word meaning and world knowledge in language comprehension. *Science* 304 438–441. 10.1126/science.1095455 15031438

[B26] Hoeks J. C. J. Stowe L. A. Doedens G. (2004). Seeing words in context: the interaction of lexical and sentence level information during reading. *Cogn. Brain Res.* 19 59–73. 10.1016/j.cogbrainres.2003.10.022 14972359

[B27] Kauhanen M. Korpelainen J. Hiltunen P. Määttä R. Mononen H. Brusin E. (2000). Aphasia, depression, and non-verbal cognitive impairment in ischaemic stroke. *Cerebrovasc. Dis.* 10 455–461. 10.1159/000016107 11070376

[B28] Kawohl W. Bunse S. Willmes K. Hoffrogge A. Buchner H. Huber W. (2010). Semantic event-related potential components reflect severity of comprehension deficits in aphasia. *Neurorehabil. Neural Repair* 24 282–289. 10.1177/1545968309348311 19861589

[B29] Keuleers E. Brysbaert M. New B. (2010). SUBTLEX-NL: a new measure for Dutch word frequency based on film subtitles. *Behav. Res. Methods* 42 643–650. 10.3758/BRM.42.3.643 20805586

[B30] Khachatryan E. van Vliet M. De Deyne S. Storms G. Manvelyan H. Van Hulle M. M. (2014). “Amplitude of N400 component unaffected by lexical priming for moderately constraining sentences,” in *Proceedings of 2014 4th International Workshop on Cognitive Information Processing*, Copenhagen, 0–5.

[B31] Kojima T. Kaga K. (2003). Auditory lexical-semantic processing impairments in aphasic patients reflected in event-related potentials (N400). *Auris Nasus Larynx* 30 369–378. 10.1016/j.anl.2003.07.007 14656562

[B32] Kuperberg G. R. Sitnikova T. Caplan D. Holcomb P. J. (2003). Electrophysiological distinctions in processing conceptual relationships within simple sentences. *Cogn. Brain Res.* 17 117–129. 10.1016/S0926-6410(03)00086-7 12763198

[B33] Kutas M. Federmeier K. D. (2011). Thirty years and counting: finding meaning in the N400 component of the event-related brain potential (ERP). *Annu. Rev. Psychol.* 62 621–647. 10.1146/annurev.psych.093008.131123 20809790 PMC4052444

[B34] Kutas M. Hillyard S. (1980). Reading senseless sentences: brain potentials reflect semantic incongruity. *Science* 207 203–205. 10.1126/science.7350657 7350657

[B35] Kutas M. Iragui V. (1998). The N400 in a semantic categorization task across 6 decades. *Electroencephalogr. Clin. Neurophysiol.* 108 456–471. 10.1016/S0168-5597(98)00023-9 9780016

[B36] Lau E. Almeida D. Hines P. C. Poeppel D. (2009). A lexical basis for N400 context effects: evidence from MEG. *Brain Lang.* 111 161–172. 10.1016/j.bandl.2009.08.007 19815267 PMC2783912

[B37] Lau E. F. Holcomb P. J. Kuperberg G. R. (2013). Dissociating N400 effects of prediction from association in single word contexts. *J. Cogn. Neurosci.* 25 484–502. 10.1162/jocn 23163410 PMC3657387

[B38] Lau E. F. Phillips C. Poeppel D. (2008). A cortical network for semantics: (de)constructing the N400. *Nat. Rev. Neurosci.* 9 920–933. 10.1038/nrn2532 19020511

[B39] Lendrem W. Lincoln N. B. (1985). Spontaneous recovery of language in patients with aphasia between 4 and 34 weeks after stroke. *J. Neurol. Neurosurg. Psychiatry* 48 743–748. 10.1136/jnnp.48.8.743 2411876 PMC1028444

[B40] Luck S. J. (2005). *An Introduction to Event Related Potential Technique. Monographs of the Society for Research in Child Development*, Vol. 79 Cambridge, MA: MIT Press, 10.1111/mono.12122

[B41] Lynch K. E. Damico J. S. Abendroth K. J. Nelson R. L. (2013). Reading performance subsequent to aphasia: strategies applied during authentic reading. *Aphasiology* 27 723–739. 10.1080/02687038.2012.748182

[B42] Marian V. Bartolotti J. Chabal S. Shook A. (2012). CLEARPOND: cross-linguistic easy-access resource for phonological and orthographic neighborhood densities. *PLoS ONE* 7:e43230 10.1371/journal.pone.0043230 PMC3423352 22916227

[B43] Swaab T. Brown C. Hagoort P. (1997). Spoken sentence comprehension in aphasia: event-related potential evidence for a lexical integration deficit. *J. Cogn. Neurosci.* 9 39–66. 10.1162/jocn.1997.9.1.39 23968179

[B44] Swaab T. Y. Brown C. Hagoort P. (1998). Understanding ambiguous words in sentence contexts: electrophysiological evidence for delayed contextual selection in Broca’s aphasia. *Neuropsychologia* 36 737–761. 10.1016/S0028-3932(97)00174-7 9751439

[B45] Tsolaki A. Kosmidou V. Hadjileontiadis L. Kompatsiaris I. Y. Tsolaki M. (2015). Brain source localization of MMN, P300 and N400: aging and gender differences. *Brain Res.* 1603 32–49. 10.1016/j.brainres.2014.10.004 25445998

[B46] van Berkum J. J. Hagoort P. Brown C. M. (1999). Semantic integration in sentences and discourse: evidence from the N400. *J. Cogn. Neurosci.* 11 657–671. 10.1162/089892999563724 10601747

[B47] van de Meerendonk N. Kolk H. H. J. Vissers C. T. W. M. Chwilla D. J. (2010). Monitoring in language perception: mild and strong conflicts elicit different ERP patterns. *J. Cogn. Neurosci.* 22 67–82. 10.1162/jocn.2008.21170 19199401

[B48] Van Petten C. (1993). A comparison of lexical and sentence-level context effects in event-related potentials. *Lang. Cogn. Process.* 8 485–531. 10.1080/01690969308407586

[B49] Van Petten C. Coulson S. Weckerly J. Federmeier K. Folstein J. Kutas M. (1999). Lexical association and higher-level semantic context: an ERP study. *J. Cogn. Neurosci. Suppl.* 1:46.

[B50] Van Petten C. Kutas M. (1990). Interactions between sentence context and word frequency in event-related brain potentials. *Mem. Cogn.* 18 380–393. 10.3758/BF03197127 2381317

[B51] Van Vliet M. Manyakov N. V. Storms G. Fias W. Wiersema J. R. Van Hulle M. M. (2014). Response-related potentials during semantic priming: the effect of a speeded button response task on ERPs. *PLoS ONE* 9:e87650 10.1371/journal.pone.0087650 PMC3916390 24516556

[B52] Verbeke G. Molenberghs G. (2000). *Linear Mixed Models for Longitudinal Data.* New York, NY: Springer.

[B53] Vidaurre C. Sander T. H. Schlögl A. (2011). BioSig: the free and open source software library for biomedical signal processing. *Comput. Intell. Neurosci.* 2011 935364 10.1155/2011/935364 PMC3061298 21437227

[B54] Wade D. Hewer R. David R. Enderby P. (1986). Aphasia after stroke: natural history and associated deficits. *J. Neurol. Neurosurg. Psychiatry* 49 11–16. 10.1136/jnnp.49.1.11 2420939 PMC1028640

[B55] Wassenaar M. Hagoort P. (2005). Word-category violations in patients with Broca’s aphasia: an ERP study. *Brain Lang.* 92 117–137. 10.1016/j.bandl.2004.05.011 15629487

[B56] Wilson K. R. O’Rourke H. Wozniak L. A. Kostopoulos E. Marchand Y. Newman A. J. (2012). Changes in N400 topography following intensive speech language therapy for individuals with aphasia. *Brain Lang.* 123 94–103. 10.1016/j.bandl.2012.06.005 22944529

