# Supplementary material for: Sentence Context Prevails Over Word Association in Aphasia Patients with Spared Comprehension: Evidence from N400 Event-Related Potential
Source: Front Hum Neurosci. 2017 Jan 10;10:684. doi: 10.3389/fnhum.2016.00684 (PMC5223168; doi:10.3389/fnhum.2016.00684)
Supplement: Supplementary file 1 [file Table_1.DOCX]

Supplementary Material

**Semantic Word-Association Processing in Sentence Context in Patients with Aphasia: An Event Related Potential Study**

**Elvira Khachatryan^,*^, Miet De Letter, Gertie Vanhoof, Ann Goeleven, Marc M. Van Hulle**

*** Correspondence:** Elvira Khachatryan, MD: [Elvira.khachatryan@kuleuven.be](mailto:Elvira.khachatryan@kuleuven.be)

# Table S1: Mean and standard deviations (between brackets) of hit-rate for each sentence group and subject group

| **Subject Group** | ***cong_HA*** | ***cong_LA*** | ***incong_HA*** | ***incong_LA*** |
| --- | --- | --- | --- | --- |
| **Young control** | 0.980 (0.02) | 0.967 (0.02) | 0.933 (0.04) | 0.976 (0.02) |
| **Older control** | 0.971 (0.03) | 0.957 (0.045) | 0.943 (0.046) | 0.982 (0.038) |
| **Aphasia group** | 0.924 (0.06) | 0.913 (0.06) | 0.775 (0.2) | 0.892 (0.15) |
