# Supplementary material for: Sentence Context Prevails Over Word Association in Aphasia Patients with Spared Comprehension: Evidence from N400 Event-Related Potential
Source: Front Hum Neurosci. 2017 Jan 10;10:684. doi: 10.3389/fnhum.2016.00684 (PMC5223168; doi:10.3389/fnhum.2016.00684)
Supplement: Supplementary file 2 [file Table_2.DOCX]

Supplementary Material

**Semantic Word-Association Processing in Sentence Context in Patients with Aphasia: An Event Related Potential Study**

**Elvira Khachatryan^,*^, Miet De Letter, Gertie Vanhoof, Ann Goeleven, Marc M. Van Hulle**

*** Correspondence:** Elvira Khachatryan, MD: [Elvira.khachatryan@kuleuven.be](mailto:Elvira.khachatryan@kuleuven.be)

# Table S2: Electrodes with significant correlations between N400 effect for each sentence group and AAT scores for patients

| *Electrode* | Comp – cong_LA | Comp – cong_HA | Nam – cong_HA | Nam – cong_LA | TT – incong_HA | TT – incong_LA |
| --- | --- | --- | --- | --- | --- | --- |
| *F3* | 0.687* |  |  |  |  |  |
| *Fz* | 0.637* |  |  |  |  |  |
| *F4* | 0.73* |  | 0.625* |  |  |  |
| *FC5* | 0.71* |  |  |  |  |  |
| *FC1* | 0.713* |  |  |  |  |  |
| *FC2* | 0.635* |  |  |  |  |  |
| *FC6* | 0.81*** | 0.71* | 0.6* |  |  |  |
| *C3* | 0.798*** |  |  |  |  |  |
| *Cz* | 0.719* |  |  |  |  |  |
| *C4* | 0.828*** |  |  |  |  |  |
| *T8* | 0.85*** |  |  |  |  |  |
| *CP5* | 0.836*** | 0.62* |  |  |  |  |
| *CP1* | 0.819*** | 0.61* |  | 0.65* |  |  |
| *CP2* | 0.815*** |  |  | 0.61* |  |  |
| *CP6* | 0.86*** |  |  | 0.64* |  |  |
| *P7* | 0.77** |  |  | 0.74** |  |  |
| *P3* | 0.711* |  |  | 0.62* |  |  |
| *Pz* | 0.772** | 0.62* |  |  |  |  |
| *P4* | 0.73* |  |  | 0.61* |  |  |
| *P8* | 0.674* |  |  | 0.62* |  |  |
| *PO3* | 0.814*** | 0.63* |  | 0.69* |  |  |
| *PO4* | 0.694* |  |  |  |  |  |
| *O1* | 0.613* | 0.67* |  |  |  |  |
| *Oz* | 0.6465* |  |  | 0.62* |  |  |
| *O2* | 0.685* |  |  | 0.66* |  |  |
| *Fp2* |  |  |  |  | -0.63* | -0.65* |
