# Supplementary material for: Sentence Context Prevails Over Word Association in Aphasia Patients with Spared Comprehension: Evidence from N400 Event-Related Potential
Source: Front Hum Neurosci. 2017 Jan 10;10:684. doi: 10.3389/fnhum.2016.00684 (PMC5223168; doi:10.3389/fnhum.2016.00684)
Supplement: Supplementary file 3 [file Data_Sheet_1.DOCX]

Supplementary Material

Sentence Context Prevails over Word Association in Aphasia Patients with Spared Comprehension: Evidence from N400 Event-Related Potential

Elvira Khachatryan*, Miet De Letter, Gertie Vanhoof, Ann Goeleven, Marc M. Van Hulle

*** Correspondence:** Elvira Khachatryan, MD: [Elvira.khachatryan@med.kuleuven.be](mailto:Elvira.khachatryan@med.kuleuven.be)

1. **Evaluation of 50 ms time windows to compare N400 onset latencies**

**Method**

Before calculating the latency for each of the subject groups (main text), we wanted to detect the difference between those latencies and ascertain that this difference (even small) is statistically significant. We concentrate on the N400 as it is our main focus. We split the time-range of 250 – 600 ms (based on visual inspection) into smaller time intervals of 50 ms and included those into the interactive model as the effect of time–window (TW – 7 levels) together with the effects of subject group (SubG) and sentence group (SG – 4 levels). We were particularly interested in the effect of a three way interaction between those factors (TW × SubG × SG), as the significance of this effect would mean that the ERP responses differed between subject groups. As we wanted to study the difference between each pair of subject groups, we conducted a separate comparison for each pair (young versus older healthy controls, young controls versus patients, older controls versus patients); hence, we considered three models, each one with a 7 × 2 × 4 structure.

**Result**

Before analyzing the N400 latencies using Luck’s method (see main text), we verified whether there is a statistically significant difference in onset latency of the N400 potential between subject groups. When comparing the two control groups, the interactive mixed design analysis of variance (ANOVA) with TW (7 levels), SubG (2 levels) and SG (4 levels) factors applied to the average EEG amplitude of 50 ms short time-windows did not show any statistically significant effect of a three way TW×SubG×SG interaction on any of the 32 electrodes. When comparing young healthy controls and patients, as expected, a statistically significant effect of TW×SubG×SG interaction was observed on all electrodes (p<0.05 in all cases), except for the very frontally located ones (Fp1, Fp2, AF3, AF4, F7 and F8). Finally, when comparing older controls and patients, the effect of TW×SubG×SG interaction was significant on all electrodes, again except for the said frontal ones.

These comparisons clearly indicate the difference in N400 shape between healthy controls and patients, which might serve as an evidence for a difference in N400 onset latency, whence, our motivation to assess latencies per subject group.
